# Antecedents of Corporate Environmental Commitments: The Role of Customers

**DOI:** 10.3390/ijerph15061191

**Published:** 2018-06-06

**Authors:** Asghar Afshar Jahanshahi, Alexander Brem

**Affiliations:** 1 CENTRUM Católica Graduate Business School, Lima 15023, Peru; 2 Pontificia Universidad Católica del Perú, Lima 15023, Peru; 3 School of Business and Economics, Friedrich-Alexander-Universität Erlangen-Nürnberg (FAU), 90429 Nuremberg, Germany; alexander.brem@fau.de; 4 Technology Entrepreneurship and Innovation (TEI), University of Southern Denmark, 6400 Sønderborg, Denmark

**Keywords:** corporate environmental commitments, customer capital, environmental collaboration, SMEs, developing country

## Abstract

The management of natural environments has become a fundamental issue for companies in recent years. A firm’s environmental commitment affects all levels of its operation. In this study, we investigated whether having an effective and constant relationship with customers over time (customer capital) makes a difference to firms with a high environmental commitment compared with less environmentally committed firms. We found support for our idea by using original survey data from 149 small and medium-sized enterprises (SMEs) in Iran (2016–2017). Furthermore, we found that customer capital enhances environmental collaboration with customers which, in turn, has a positive impact on the firm’s environmental commitments. These findings provide empirical evidence for the important role of “getting closer to customers” as a way of enhancing corporate environmental responsibility in developing countries with weak institutional environments.

## 1. Introduction

Industrial units have been considered as a major source of environmental degradation [1]. Therefore, environmental preservation by firms has gained the increasing attention of scholars in different fields in recent years [2,3,4]. Earlier research in this area posed an interesting question in this context: what makes firms who are highly committed to protecting the natural environment different from less environmentally committed firms in developing countries with weak institutional environments? [5]. Executive awareness of environmental issues is one of the primary steps for increasing a firm’s environmental responsibility [6,7,8]. Therefore, in order to have a better understanding of the origin of corporate environmental commitments, we need to identify those activities that enhance the generation and accumulation of environmental information inside organizations.

Customers’ awareness about environmental problems and issues has increased in recent years [9] and they are showing more interest in buying environmental-friendly products even at higher prices [10]. Being closer to customers and having effective interactions with them—described as customer capital [11,12]—provides valuable opportunities for firms to know customers better and understand their environmental concerns. Such closeness and interaction enhance customers’ collaboration in different areas, such as finding way to reduce or prevent air, land, and water pollution by firms. 

This paper contributes to the current literature by showing the important role of customer capital in enriching corporate environmental commitments in the context of developing countries. In the context of developed countries, governmental regulations contribute significantly to the environmental performance of a company [13]. According to previous studies, governmental command-and-control environmental regulations, such as industrial pollution control, often perform poorly in most developing countries [14]. In these types of countries, a corrupt and inefficient system of government makes it easier for firms to stay off the legal radar screen [15,16,17]. Additionally, a low level of political and social trust in these countries is another important factor that encourages firms to avoid responsible business practices [18,19]. Our empirical evidence from 149 Iranian SMEs revealed that taking critical steps to address a host of environmental issues is more common among firms that are closer to their customers. The durable relationships that a firm builds with its customers over time enhance the environmental information available within firms and make them more responsible for the surrounding natural environment.

## 2. Literature Review and Hypothesis Development

### 2.1. Importance of Corporate Environmental Commitments

Corporate environmental commitments exhibit the level of organizational interest for effectively managing its business and natural environments [20]. Some companies are taking more proactive strategies and showing higher commitment to protecting their surrounding natural environment by minimizing water and energy wastage, and preventing air and land pollution, compared to other companies [21]. Such proactive strategies help firms to differentiate their product and service in the market, improve their company image [22] and public health [23]. Finally, these strategies generate competitive advantages, depending on other external factors [20]. Several organizational and institutional factors motivate or force firms to incorporate environmental issues into their daily business activities and practices such as governmental regulations or the increasing costs of pollution control [24], shareholder pressure [25], neighborhood and community group forces [26] and customer pressure [27].

According to Baughn et al. [28], Welford [29] and Kimber et al. [30], Asian countries usually have different political systems, regulatory regimes, social context and cultural norms compared to other nations in the world. Apparently, this has an effect on a firm’s environmental responsibility and commitments. For instance, in most developing countries in Asia, such as Iran, the business environment suffers from weak and underdeveloped governmental regulations and standards. Due to ineffective environmental laws and governance, private companies feel less (or even no) pressure to follow basic environmental protection standards. In such contexts, customer demands play an important role in persuading firms to take responsibility for the natural environment [26]. In this regard, by using data from 142 Iranian firms in the food industry, Hosseininia and Ramezani [31] found that customers are a major factor for improving the sustainable performance of Iranian SMEs.

### 2.2. Customer Capital and Corporate Environmental Commitments

Customers not only act as the engine for companies’ development and growth [32], but they also play a key role in shaping the companies’ behavior in the marketplace [33]. In recent years, demands for environmentally friendly business activities and practices have increased significantly in line with the rise of customers’ environmental consciousness [6]. Hence, firms need to be closer to their customers to better realize and understand their environmental concerns.

Customer capital is the outcome of an organization’s lifetime relationship with its customers [34]. Therefore, the more interaction an organization has with its customers, the more possibilities it has to develop customer capital [35]. As a consequence, customer capital keeps managers informed about customers’ preferences [32]. It enables managers to better understand their customers’ perspectives, expectations, beliefs, and values on environmental issues [36]. In this paper, we expect that those firms with a strong ability to build long-term relationships with customers will show more commitment to protecting the natural environment. These insights led to our first hypothesis:

**Hypothesis** **1.** *There is a positive relationship between the firms’ customer capital and their environmental commitments*.

### 2.3. The Mediating Role of Environmental Collaboration with Customers

Emphasizing customer capital provides an opportunity for firms to build a close relationship with customers [37,38]. As a result, we can expect a higher level of information acquisition and sharing regarding environmental issues from the customers [39]. Most importantly, developing a reciprocal relationship with customers enhances the possibility of their participation [40] and earning their trust [41]. Collaboration with customers raises managers’ awareness about the business environment and reduces the possibility of making decisions in a vacuum, without considering the positive and negative potential effects on the surrounding environment [42]. Environmental collaboration with customers and other stakeholders can improve the firm’s commitment to the natural environment. Within such a rich collaborative environment, customers can help firms with the selection of pollution prevention technologies [43,44]. Therefore, in the second hypothesis we expected that getting closer to customers and building an ongoing relationship with them will lead to joint planning and decision making regarding environmental issues, which eventually improves corporate environmental commitments.

**Hypothesis** **2.** *Environmental collaboration with customers mediates the relationship between customer capital and their environmental commitments*.

## 3. Methodology

### 3.1. The Context of the Study

The empirical context of this research is small and medium-sized enterprises in Iran. In terms of the environmental performance index, Iran ranked 83 out of 178 countries in the world in 2017. Iranians are prone to suffer from serious health effects from air, water and land pollution, according to recent World Health Organization (WHO) reports. According to Iran’s most recent official statistics, every two hours, one person dies of pollution-related causes in Tehran, on average. In order to minimize industrial pollution, more recently, the government tried to force companies to meet environmental standards and regulations. However, the country has been ranked 131 out of 176 in the Corruption Perception Index (CPI) in 2017—the year we did the survey. In such a business environment, finding a way to avoid implementing costly environmental protections standards is not difficult. Hence, we think Iran is an ideal case to research this topic.

### 3.2. Sample and Data Collection

We tested our hypotheses by collecting survey data (2016–2017) from small and medium-sized enterprises (SMEs) in the south-east of Iran. There is no well-accepted definition for SMEs in Iran. Usually firms with more than 10 and fewer than 50 employees are referred to as SMEs in Iran [45]. According to the Central Bank of Iran, over 90% of registered firms in Iran are small and medium sized [46]. In total, 175 SMEs received our survey. We obtained the list of SMEs from three government databases, including the Kerman Industrial Estate (http://iec.kr.ir/), Fars Industrial Estate (http://www.farsiec.ir/), and Hormozgan Industrial Estate (http://www.hriec.ir/). In each SME, environmental protection or sales managers and marketing managers filled out the survey. In this study the unit of analysis is the firm level. 

We have used a back translation method for translating the original survey items to Persian [47]. In doing so, one translator (not authors) fluent in both English and Persian translated the original English survey into Persian, and another translator independently back-translated the Persian survey into English [47,48]. One of the authors had a meeting with two translators to compare the original version of the survey with the back translated one. Any confusing, unclear, and inappropriate words or items were modified. Before launching the final questionnaire, we conducted in-depth interviews with five top-level managers (three CEOs, one finance manager and one human resource manager), wherein we asked these managers to identify ambiguity and wording format in order to confirm the appropriateness of our measurement items. The questionnaires were personally dispatched to and collected from each respondent after the scheduled time period (almost a week). The participants were guaranteed absolute anonymity in order to avoid socially desirable responses in the survey [49].

Among 175 participants, 149 managers from 149 SMEs fully completed all the items resulting in a response rate of 85%. In the final sample, 88 responses were from environmental managers, and 61 responses were from sales and marketing managers. The average age of the respondents (environmental protection or sales and marketing managers) was 42.13 years (S.D. = 7.63), the average firm age was 15.54 years (S.D. = 7.83), and the average number of employees or firm size was 17.97 (S.D. = 11.66). In addition, 67% of the respondents held academic degrees, and were mostly men (71.8%). In terms of industry affiliation, 22.1% of the SMEs were from the service sector; 16.8% from manufacturing; 16.1% from the retail trade; 13.4% from agriculture; 10.7% were wholesalers; 8.1% from transportation, communications and public utilities; 8.1% from finance, real estate and insurance; and about 4.7% from construction.

The Harman one-factor test was conducted [50] to check the possibility of common method variance in our survey data. In doing so, we used factor analysis of measures related to customer capital, environmental collaboration with customers and corporate environmental commitment. The first factor explained 20.1% of total variance. Therefore, no single factor emerged, and no single factor accounted for the majority of variance [50,51]. For reducing the common method variance [52], the order of measurement items was randomized as well. Finally, we compared early respondents (first 25% of participants) with late respondents (last 25% of participants) on both firm size and age; we did not find any significant difference. This indicates that it is unlikely that nonresponse bias affected our data and results [53].

### 3.3. Measures

#### 3.3.1. Corporate Environmental Commitments

According to Henriques and Sadorsky [25], corporate environmental commitments refer to “an organization-wide recognition of the importance of the natural environment that influences organizations to act in ways consistent with the interests of the natural environment”. Corporate environmental commitments were measured by eight items from [54]. On a five-point Likert scale (1 = very rare, 5 = very common), respondents described how their organization has (1) committees dedicated to deal with environmental issues, (2) a formal plan for dealing with environmental issues, (3) formal documents describing an environmental plan, (4) manuals detailing environmental procedures, (5) employee training programs on environmental procedures, (6) employees whose job is to deal with environmental issues, (7) a reward system that recognizes environmental achievements, and (8) environmental information in external communications. The Cronbach’s value of the scale of corporate environmental commitments was 0.88.

#### 3.3.2. Customer Capital

Customer capital refers to the informal and formal relationships that a firm has with its customers over time [55]. In this paper, customer capital was assessed using the four items developed by [55]. We asked the respondents, “With respect to your main competitors indicate the degree in which your company reached the following objectives (1 = strong down and 7 = strong up)”: 1. Improvement of quality; 2. The level your customers recommend your company; 3. Recurrence of purchases; and 4. Good reputation and prestige. The Cronbach’s alpha index for customer capital was 0.87.

#### 3.3.3. Environmental Collaboration with Customers

Environmental collaboration with customers refers to the inter-organizational interactions between the management and its major customers, including several aspects, such as joint environmental goal setting, and working together to reduce pollution or other harmful environmental impacts [56]. Based on [56], we used 5 items to measure firms’ environmental collaboration with customers on a seven-point Likert scale from 1, not at all, to 7, to a great extent. We asked respondents: “During the past two years, to what extent did your company engage in the following environmental activities with your major customers?” The five items are: “Achieving environmental goals collectively”, “Developing a mutual understanding of responsibilities regarding environmental performance”, “Working together to reduce the environmental impact of our activities”, “Conducting joint planning to anticipate and resolve environmental-related problems”, and “Making joint decisions about ways to reduce the environmental impact of our product”. The Cronbach’s alpha index for this scale was 0.97.

#### 3.3.4. Control Variables

In order to check whether our main model is robust, we added individual and firm-level control variables to our analyses. In the Iranian context, previous studies show that a manager’s age, education and gender play an important role in a firm’s engagement in environmentally oriented actions [31,45,57]. At the individual level, we controlled respondents age, gender, and education (1 = High school; 2 = Attended college; 3 = Undergraduate; 4 = Attended graduate school; 5 = Master’s; 6 = Attended doctoral program; 7 = Doctorate). Firm size and age may have an effect on an organization’s tendency to act in an environmentally responsible manner [7,58]. Therefore, at the firm level, we controlled the firm size by adding the total number of employees [59] and firm age, adding the number of years a company has been in existence [60]. Finally, previous studies have highlighted the important role of industry for a firm’s environmental and social commitments [25,61]. Hence, we included the firm’s primary industry type ((1) service sectors, (2), manufacturing (3), retail trade (4), agriculture (5), wholesale trade (6), transportation, communications and public utilities (7), finance, real estate and insurance, and (8) construction) as a control variable in our analysis. The firms participating in our survey represented 48 four-digit Standard Industrial Classification (SIC) codes.

## 4. Findings

We used SPSS (24) software for analyzing our data and testing our hypotheses. Correlations, means, and standard deviations for all measured items are reported in Table 1.

Before testing the hypotheses of the study, we checked the variance inflation factors (VIF) to exclude multicollinearity. The highest VIF was 1820, proving that multicollinearity was not a problem in our research [62]. As can be seen in Table 1, there are positive and significant correlations between independent and dependent variables. The first hypothesis predicted a positive relationship between firms’ customer capital and corporate environmental commitment. As shown in Model 2 in Table 2, the relationship between customer capital and corporate environmental commitments is positive and statistically significant (β = 0.278, *p* < 0.01). Thus, Hypothesis 1 was fully supported.

In the second hypothesis, we expected that environmental collaboration with customers would mediate the relationship between customer capital and corporate environmental commitment. We used the regression procedures suggested by [63] to test for the mediating effect of environmental collaboration. According to [63], the mediated regression has to meet three requirements. The first and second independent variables (customer capital) should relate significantly to the dependent variable (corporate environmental commitments) and to the mediator (environmental collaboration with customers). The last requirement, when environmental collaboration with customers is entered before the customer capital, the significance of the relationship of the independent variable (customer capital) to the dependent variable (corporate environmental commitments) should decrease if environmental collaboration with customers is the mediator. By using hierarchical regression analysis in Model 2 in Table 2, we found a positive and significant relationship between firms’ customer capital and corporate environmental commitment (β = 0.278, *p* < 0.01). So, the first requirement of the mediation model was satisfied. As we can see in Table 2, Model 3, customer capital (β = 0.784, *p* < 0.001) is positively and significantly related to environmental collaboration with customers, which satisfies the second requirement of the mediation model.

In the last step, we entered both independent and mediator variables and corporate environmental commitments as a dependent variable. As can be seen in Table 2, Model 4, the significance of the relationship of the independent variables (customer capital) to the dependent variable (corporate environmental commitments) decreases and become non-significant (β = −0.098, n.s.) when environmental collaboration with customers is the mediator. This supports the last requirement of the mediation model. Therefore, the second hypothesis was fully supported.

## 5. Discussion

In most developing countries, environmental protection standards are weak and underdeveloped. Furthermore, a wide variety of illegal and corrupt behaviors are common practice in many firms. In business environments which are characterized by high levels of corruption, firms perceive less pressure to strictly implement costly environmental protection standards in their operational processes [17]. Recent studies reveal that increasing customer environmental awareness may motivate firms to show more commitment to protecting the natural environment [6]. Previous studies showed that Iranian customers have an acceptable level of environmental awareness [64].

Having relevant information plays a crucial role in corporate managers’ awareness of environmental problems [65]. Such awareness is a primary step for enhancing corporate environmental commitments [5,57,66]. In order to understand which factors contribute to the firms’ environmental commitments, we need to concentrate on the factors that enrich a firm’s environmental consciousness. Therefore, the main purpose of the paper was to identify firms that show more commitment to the preservation of the natural environment by considering the role of customers. Our survey data from 149 SMEs reveals that the extent to which firms builds an informal and formal relationship with its customers (customer capital) can strengthen corporate environmental commitments. Each customer has different ideas and concerns about environmental issues, such as air and water pollution, environmental degradation, and the waste disposal produced by firms. Getting closer to the customers provides an opportunity for firm managers to better understand the environmental issues. 

## 6. Conclusions

Previous studies in the Iranian context highlighted the importance of employees in improving the level of firms’ environmental and social responsibility [67]. Our results in the Iranian context revealed that establishing ongoing relationships with customers enhances environmental collaboration with customers, which in turn, influences the corporate environmental commitments. The firm’s constant relationship with customers facilitates joint environmental goal setting and working together to reduce air and water pollution or other harmful environmental impacts [56]. Through environmental collaboration with customers, firms are in a better position to design and produce its products in compliance with the customers’ environmental expectations [68]. These days, there are also tools available that support such a process. A commonly mentioned tool is referred to as Responsible Research and Innovation (RRI). Here, the idea is that the external environment, and especially the customer, is integrated into the whole research or product development process, for example, through anticipation, engagement and reflection. This concept argues that companies can also reach different maturity levels, which may help organizations to integrate such an approach over time [69].

Our research suffers from several limitations, which offer fruitful avenues for future studies. The first set of research limitations is connected to the generalizability of our results. We only surveyed SMEs that operate in a specific region, that is, in three provinces (Kerman, Fars and Khuzestan) in south-east Iran. This may decrease the generalizability of our results. It is advisable that future researchers test the ideas developed in our study across different settings and regions. The sample of this study is only SMEs because over ninety percent of firms in Iran belong to this sector, hence, future research might the same relationships using data from large-sized or multinational companies. We tested our hypotheses by using cross-sectional data. Results of this study provide the foundation to form and test specific causal relationships. In doing so, a longitudinal design would be beneficial. According to Hope and Jones [70] and Rice [71], the religious beliefs of managers may impact on their attitudes to environmental issues. In this study, we had a homogeneous sample in terms of religion (all the participants were Muslim and Shia). Future research may test the relationship between the variables of the study with a religiously heterogeneous sample. Lastly, among the four major stakeholders (social and non-social stakeholders, employees, customers, and government [72]) which may affect the level of a firm’s environmental commitment, we have only considered the effects of customers. Our study depicts only a partial picture of the factors that drive firms’ environmental commitments. To achieve a more comprehensive picture, future research should consider all of the influential factors.

## Figures and Tables

**Table 1:** Correlation and Descriptive Statistics.

	Mean	S.D.	1	2	3	4	5	6	7	8
1, Age	42.13	7.63	1.00							
2, Education	3.56	1.66	−0.06	1.00						
3, Gender	1.72	0.45	0.08	0.01	1.00					
4, Firm size	15.54	7.83	0.15	0.01	0.05	1.00				
5, Firm age	17.97	11.66	0.09	0.00	0.03	0.15	1.00			
6, Industry	4.79	2.58	0.00	0.12	−0.09	−0.04	−0.03	1.00		
7, Customer Capital	3.06	0.90	0.16	0.16	−0.03	−0.02	0.13	−0.06	1.00	
8, Environmental collaboration	4.17	1.49	−0.11	0.377 **	−0.02	−0.03	0.05	0.00	0.513 **	1.00
9, Environmental commitment	4.02	1.32	−0.04	0.429 **	0.03	0.10	0.207 *	−0.03	0.302 **	0.488 **

* Correlation is significant at the 0.05 level (2-tailed). ** Correlation is significant at the 0.01 level (2-tailed).

**Table 2:** Direct and indirect effect of customer capital on corporate environmental commitments.

Variables Entered	Model 1	Model 2	Model 3	Model 4
	Commitment	Commitment	Collaboration	Commitment
Manager’s Age	−0.010	−0.011	−0.034 *	0.001
Education	0.360 ***	0.344 ***	0.264 ***	0.296 ***
Gender	0.170	0.138	0.080	0.160
Firm Size	0.011	−0.007	−0.010	0.000
Firm Age	0.022 **	0.012	−0.002	0.017 *
Industry	−0.032	−0.031	0.013	−0.029
Customer Capital		0.278 **	0.784 ***	−0.098
Env. Collaboration				0.338 ***
*R* ^2^	0.246	0.290	0.389	0.393
Adj. *R*^2^	0.210	0.251	0.354	0.352
F	6.954 ***	7.310 ***	10.933 ***	9.485 ***

Note. * *p* < 0.05, ** *p* < 0.01, *** *p* < 0.001.
